# Validation of the ProVent mortality prediction model in patients receiving ≥7 days of mechanical ventilation

**DOI:** 10.1093/annalsats/aaoag213

**Published:** 2026-07-25

**Authors:** Ye Eun Grace Jung, Ellen Caldwell, Kelly C Vranas, Catherine L Hough

**Affiliations:** Division of Pulmonary Sciences, Allergy and Critical Care Medicine, University of Colorado, Aurora, CO, United States; Division of Pulmonary, Allergy, and Critical Care Medicine, Department of Medicine, Oregon Health & Science University, Portland, OR, United States; Division of Pulmonary, Allergy, and Critical Care Medicine, Department of Medicine, Oregon Health & Science University, Portland, OR, United States; Center to Improve Veteran Involvement in Care, VA Portland Health Care System, Portland, OR, United States; Division of Pulmonary, Allergy, and Critical Care Medicine, Department of Medicine, Oregon Health & Science University, Portland, OR, United States

To the Editor,

Approximately 790 000 people in the United States develop acute respiratory failure requiring mechanical ventilation (MV) annually. Nearly 40% of these patients receive ≥4 days of MV (ie, prolonged MV).^1^ These patients experience high mortality and morbidity, both in-hospital and after discharge, with fewer than 10% to 20% of patients ever returning home.^1^^,^^2^

Despite the growing prevalence of patients requiring prolonged MV, prognostication of long-term outcomes among these patients is challenging and represents a barrier to shared decision-making with their families and clinicians.^3^ Prolonged MV models (ProVent 14 and ProVent), developed to tackle this issue, have been shown to accurately predict 1-year mortality in patients receiving MV for 14 and ≥21 days, respectively, using readily available clinical variables.^4^^,^^5^ However, it is unknown how these models perform for patients receiving MV for shorter durations. Since many treatment decisions are made before day 14 of MV, we sought to evaluate whether ProVent could accurately predict 90-day and 1-year mortality among patients on day 7 of MV.

## Methods

We performed a secondary analysis of the NHLBI PETAL Network’s Reevaluation of Systemic Early Neuromuscular Blockade (ROSE) study using de-identified, publicly available data. Patients were enrolled in ROSE from 48 US hospitals if they met criteria for moderate-to-severe acute respiratory distress syndrome (ARDS). Detailed characteristics of patients in ROSE are described elsewhere.^6^

We included subjects who received ≥7 days of MV and had known vital status at 1-year. We extracted ProVent model predictor variables measured at day 7 of MV (age, trauma diagnosis, platelet count, hemodialysis and/or vasopressor use). The primary outcome was 1-year mortality. Because we did not have day of death beyond day 90 for this cohort, we assessed 90-day survival as a secondary outcome.

We used descriptive statistics as appropriate, presenting data as mean (SD). Categorical variables are presented as proportions. Day 7 ProVent model predictor variables were used to compute the ProVent day 7 score, stratified as a categorical variable. The ProVent score was used in logistic regression models with 1-year survival as the primary outcome. We assessed model discrimination by the area under the receiver operating characteristic curve (AUC), and calibration using the Hosmer-Lemeshow goodness-of-fit statistic, comparing observed-to-predicted mortality in each ProVent Day 7 strata. We then compared model discrimination between ProVent 7 and APACHE III for prediction of 12-month mortality. All analyses were performed using R (v4.5.0), SAS version 9.4 (SAS Institute Inc.), and MedCalc version 23.1.7 (MedCalc Software Ltd). Preliminary data were previously presented as a poster.^7^

## Results

Among 1006 ROSE participants, 558 received ≥7 days of MV; all but 17 had outcome data (n = 541). Mean age was 56 (SD 14.7); 7% were admitted for trauma. On day 7, 24% had thrombocytopenia, 31% received hemodialysis, and 31% received vasopressors (Table 1). One-year mortality was 44%, with significant differences in survival probability when stratified by ProVent scores, which was also seen at 90 days (Figure 1). A total of 216 deaths occurred within the first 90 days, with 22 additional deaths between months 4 and 12. On day 7 of MV, the ProVent model demonstrated modest discrimination (AUC, 0.72; 95% CI, 0.68-0.76) and excellent calibration (Hosmer-Lemeshow goodness-of-fit statistic: 1.3; *P* = .73; observed/expected 1.00). Calibration was particularly good at higher ProVent scores; participants in ProVent groups 5 and 6 had 12-month mortality odds of 17.5 (95% CI, 7.5-40.8). ProVent predictor variables assessed at day 7 of MV demonstrated a statistically significant improvement in discriminative performance for 1-year mortality prediction compared with APACHE III (AUC, 0.65; 95% CI, 0.61-0.69); the difference in AUC was 0.06 (95% CI, 0.007-0.12).

**Figure 1:**
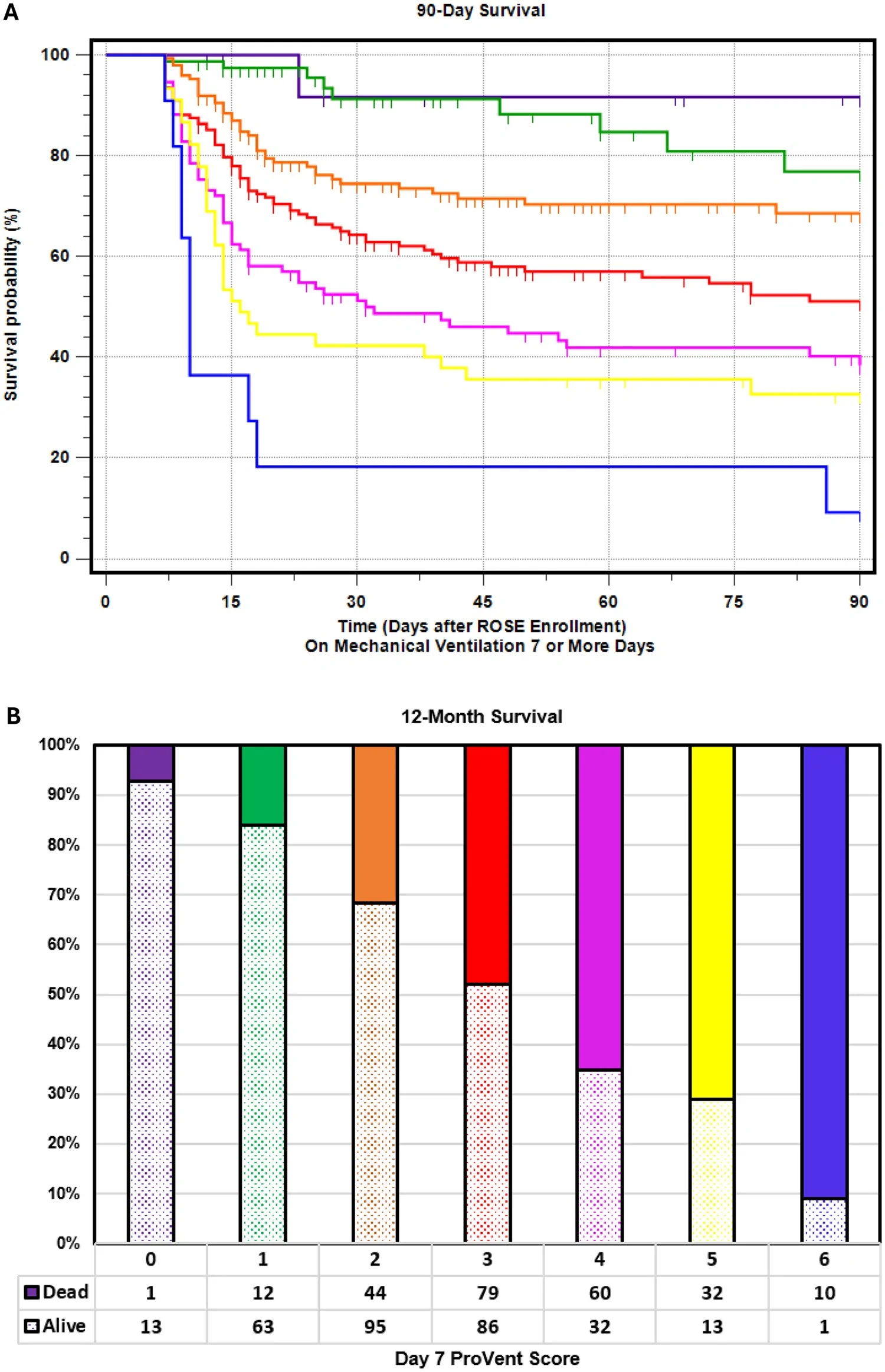
Survival by day 7 ProVent score. (A) Kaplan–Meier estimates of 90-day survival following ROSE enrollment among patients receiving invasive mechanical ventilation for ≥7 days, stratified by Day 7 ProVent score. Patients were censored at the time of last known follow-up. (B) Twelve-month survival status by Day 7 ProVent score. Hatched bar segments indicate patients alive at 12 months, and solid bar segments indicate patients who died within 12 months. Colors are consistent across panels and represent identical ProVent score categories with lines (A) and bars (B).

**Table 1:** Patient demographics at day 7 and at 1-year.

	Received ≥7 days mechanical ventilation (n = 558)	1-year mortality available (n= 541)	1-year	
			Dead (n = 238)	Alive (n = 303)
Sex, female, n (%)	258 (46)	250 (46)	114 (48)	136 (45)
Race, n (%)				
Native American	7 (1)	7 (1)	0 (0)	7 (2)
Asian	11 (2)	11 (2)	9 (4)	2 (1)
Black	78 (14)	78 (14)	33 (14)	45 (15)
White	393 (70)	378 (70)	169 (71)	209 (69)
Unknown	70 (13)	68 (13)	27 (11)	41 (14)
Ethnicity, n (%)				
Hispanic	64 (11.5)	59 (11)	24 (10)	36 (12)
Unknown	36 (7)	36 (7)	13 (6)	23 (8)
ProVent variables				
Mean age (SD)	56 (15)	56 (15)	60 (14)	53 (15)
Trauma diagnosis, n (%)	38 (7)	38 (7)	11 (5)	27 (9)
Platelets ≤ 100 × 10^9^/L, n (%)*	133 (24)	131 (24)	87 (37)	44 (15)
Receipt of renal replacement therapy, n (%)	167 (30)	166 (31)	88 (37)	78 (26)
Vasopressor use, n (%)	168 (30)	165 (31)	109 (46)	56 (19)
Median ProVent Day 7 Score (IQR)	3.0 (2.0-4.0)	3.0 (2.0-4.0)	3.0 (3.0-4.0)	2.0 (1.3-3.0)
APACHE III Score (SD)**	104 (28)	104 (28)	112 (28)	98 (27)

Abbreviation: IQR, interquartile range.

* Platelets not measured on day 7 in 32 patients; for ProVent Score, missing data were assumed normal.

** APACHE III Total Score available in 512 patients (499/541, 224/238, 275/303).

## Discussion

In this secondary analysis of a clinical trial among patients with moderate-to-severe ARDS, we found that Provent 7 was useful in predicting mortality for patients on prolonged MV for >7 days and performed modestly better than APACHE III. Furthermore, the ProVent score’s simplicity, relying on five routinely collected clinical variables, compared with APACHE’s > 20 ­variables, enhances its practical utility for clinicians and captures a patient’s accumulated organ dysfunction and early trajectory in a way that day 1 physiology (used by APACHE III) may not.

Additionally, ProVent 7 may facilitate more informed clinical decision-making earlier on during a patient’s intensive care unit (ICU) course. Prior literature has shown the incidence of death before day 8 of ICU admission among patients with chronic critical illness, of which prolonged MV accounted for 72%, increased steadily with age, underscoring the need for earlier prognostication.^8^ Moreover, evidence suggests that goals-of-care discussions addressing major treatment decisions, such as tracheostomy placement, should be happening before day 14, in part due to the fact that these discussions are iterative processes that often occur over several days.^9^ Finally, studies have shown that patients and surrogate decision makers in the ICU prefer that their clinicians provide clear, honest prognostic information and treatment options, but that this information is not always easily available.^10^ By validating the ProVent model with variables measured at day 7 instead of day 14 or beyond, this study helps to address this gap, enabling clinicians to prognosticate at an earlier time point, thereby facilitating earlier goals-of-care planning for this critically ill patient population.

Our study has several limitations. First, we tested the model in a cohort of patients with moderate-to-severe ARDS who were enrolled in a clinical trial, potentially limiting generalizability. Second, the ROSE cohort lacked date of death beyond day 90 and provided vital status at day 365, limiting the ability to represent survival curves beyond day 90, though not the overall mortality prediction. Finally, we selected APACHE III as the reference comparator as it has been validated for 1-year mortality prediction^11^ and used in prior ProVent studies^4^^,^^12^; however, comparison should be interpreted cautiously as APACHE III was neither originally designed for long-term prediction nor assessed at the same timepoint as ProVent 7. Finally, the ProVent model lacks the ability to predict other patient-centered outcomes such as long-term disability.

## Interpretation

Our findings demonstrate that the ProVent 7 model, which relies on simple, readily available variables, showed modest discrimination and excellent calibration for predicting 1-year mortality at day 7 of MV.
